# Health Impact Index. Development and Validation of a Method for Classifying Comorbid Disease Measured against Self-Reported Health

**DOI:** 10.1371/journal.pone.0148830

**Published:** 2016-02-05

**Authors:** Geir Fagerjord Lorem, Henrik Schirmer, Nina Emaus

**Affiliations:** 1 Department of caring and health science, Faculty of health sciences, The Arctic University of Norway, Tromsø, Norway; 2 Department of clinical medicine, Faculty of health sciences, The Arctic University of Norway, Tromsø, Norway; 3 Division of Cardiothoracic and Respiratory Medicine, University Hospital of Northern Norway, Tromsø, Norway; Yokohama City University, JAPAN

## Abstract

The objective of this study was to develop a method of classifying comorbid conditions that accounts for both the severity and joint effects of the diseases. The Tromsø Study is a cohort study with a longitudinal design utilizing a survey approach with physical examinations in the Tromsø municipality from 1974 to 2008, where in total 40051 subjects participated. We used Tromsø 4 as reference population and the Norwegian Institute of Public Health (FHI) panel as validation population. Ordinal regression was used to assess the effect of comorbid disease on Self-Reported Health (SRH). The model is controlled for interaction between diseases, mental health, age, and gender. The health impact index estimated levels of SRH. The comparison of predicted and observed SRH showed no significant differences. Spearman’s correlation showed that increasing levels of comorbidity were related to lower levels of SRH (R_S_ = -0.36, p <.001). The Charlson Comorbidity Index(CCI) was also associated with SRH (r = -.25, p <.001). When focusing on only individuals with a comorbid disease, the relation between SRH and the Health Impact Index (HII) was strengthened (r = -.42, p <.001), while the association between SRH and CCI was attenuated (r = -.14, p <.001). CCI was designed to control for comorbid conditions when survival/mortality is the outcome of interest but is inaccurate when the outcome is SRH. We conclude that HII should be used when SRH is not available, and well-being or quality of survival/life is the outcome of interest.

## Introduction

The prevalence of coexisting chronic conditions is rising [1]. Comorbidity is associated with worse health outcomes, more complex clinical management and increased health care costs [2]. However, how do we establish information about the impact of comorbid disease on general health status? Comorbid disease is a problem in longitudinal designs. In clinical studies of high-risk groups such as disease groups with high prevalence of comorbidity, excluding patients that develop comorbid disease will be a design weakness, especially when comorbidity is relevant for care [3].

There are several different methods to measure comorbid impact on health that utilize mortality as the preferred outcome of interest [4–7]. They were developed as methods to deal with comorbidity in prospective studies but are also applied by health planners and policy makers to estimate risk and future health costs, to assure quality in health services [6, 8, 9]. The Charlson Comorbidity Index (CCI) is the most extensively studied. The CCI identifies diseases associated with higher mortality rates but does not reflect health behaviors, resources or ability to cope with health threats. However, a recent UK study concludes that Self-Reported Health (SRH) and several other self-reported variables significantly outperformed the CCI [10]. It is a methodical weakness if the existing methods to deal with comorbidity in epidemiological studies fail to identify and correctly weigh the health impact of different diseases [11–16].

SRH is well a known, independent predictor of future health outcomes, health service use and mortality even in populations with a small or no known disease burden [10, 17]. The association between perceived health status and future health outcomes suggests a close relation between perceived health status and more objective measures of health status [14, 15]. SRH thus helps to identify at-risk individuals and illuminate underlying illnesses that may otherwise go undetected during routine examinations in both healthy and patient populations [14–16]. SRH is also related to personal, socio-environmental, behavioral and psychological factors such as coping resources, psychological strain, and physical fitness [16, 18–20]. It is proven stable across cultures, communities and different age groups [11, 13, 21–28]. SRH thus seems to go beyond a model of health as absence of disease, and several authors argue that SRH reflects comorbidity better than any additive measure of diseases [29–31]. Many studies of SRH that focus on comorbidity or more objective biological health measures are performed in patient populations. The Tromsø study allows us to estimate the impact of comorbid diseases in a general population, utilizing a survey approach and physical examinations in large representative samples of the Tromsø population [32].

We aim to use data from the Tromsø study to investigate how differences in gender, age, mental health symptoms and specific medical conditions relate to different levels of SRH. We also aim to develop and validate a new method of classifying comorbid conditions that accounts for both the severity and joint effects of the diseases. Both aims were achieved and are reported in this article.

## Material and Methods

### Sample and design

The Tromsø Study consists of six surveys conducted in the municipality of Tromsø from 1974 to 2008 [32]. The study population was recruited from all inhabitants in specific age groups in the city. The aim has been to include large, representative samples of the Tromsø population, with the invitation of whole birth cohorts and random samples. A total of 40051 participants gave informed signed consent and attended up to six separate health examinations. The attendance rate was high (66–75%). We collected samples from the Tromsø studies conducted in 1994/1995 (the Tromsø 4 panel) and 2001/2002 (the FHI panel). Our samples were drawn from the general population. They did not only include persons already suffering from a disease but also healthy subjects [32].

We used Tromsø 4 as the reference population. All inhabitants in Tromsø municipality 25 years or older were invited. The attendance rate was 69.6% for men and 74.9% for women. We excluded all that missed SRH (n = 39). The reference population thus included 12,661 men and 14,023 women aged 25–97.

Tromsø 5 invited two groups. The primary group consisted of all who had participated in the special study in 1994–95 and a smaller group (n = 1916) whom the Norwegian Institute of Public Health (FHI) invited as part of its nationwide health study (the FHI panel). We used the FHI panel as validation population. We excluded all subjects that participated in Tromsø 4 (n = 1112) to ensure independence. The validation population thus included 442 women and 362 men aged 30–79.

### Measurements

The participants completed a self-administered questionnaire that included questions on a wide range of diseases and symptoms, health behavior, social conditions, education, financial difficulties and level of physical activity. We considered three groups of health-related variables: (1) self-reported health, (2) specific medical conditions, and (3) mental health.

#### Self-reported health

The independent variable self-reported health was reported by answering the survey question ‘What is your current state of health?’ with answers ranging from poor (1) to very good (4).

#### Specific medical conditions

The conditions were self-reported by answering survey questions as “Do you have or have you had….?” We classified each participant for each known diagnosis. We included all available specific medical conditions to avoid missing possible long-term after-effects on SRH. The conditions therefore include acute diseases (e.g. myocardial infarction), chronic conditions (e.g. epilepsy) as well as conditions that had been completely resolved at the time of the survey (e.g. kidney stone). All conditions are listed in Table 1. Only participants older than 70 years answered questions about Parkinson’s disease, arthritis, rheumatoid arthritis, urinary incontinence, glaucoma, and cataract.

**Table 1:** Percentage of patients entered for each SRH category and odds ratios from the ordinal regression models by comorbid disease in the reference population (Tromsø 4).

	Self-Reported Health			Impact on Self-Reported Health			
	Count	Mean^a^	SD.	Unadjusted models	Base model	Mental conditions	Full con- textual model^d^
**Age 10-year groups**							
25–29	3046	3.14	.64	1.00^e^			1.00 ^e^
30–39	6769	3.07	.64	1.29			1.28
40–49	6644	2.88	.65	2.30			2.14
50–59	4413	2.70	.67	4.15			3.46
60–69	3099	2.49	.64	7.48			5.84
70–79	2145	2.38	.68	10.08			6.47
>80	607	2.27	.64	14.19			8.78
**Gender**							
Female	14023	2.79	.71	1.00 ^e^			1.00 ^e^
Male	12661	2.88	.68	.76			1.04
**Mental health**							
No symptoms	2061	3.22	.71	1.00 ^e^		1.00	1.00 ^e^
Some symptoms	15751	2.92	.64	2.73		2.89	2.97
Sub-threshold symptoms	4964	2.59	.65	7.12		6.93	7.66
Significant symptoms	1762	2.21	.67	21.63		17.65	20.79
**Myocardial and vascular**							
Angina	1097	2.09	.56	8.89	4.94	4.71	2.77
Myocardial infarction	758	2.21	.62	5.67	2.85	3.03	2.09
Cerebrovascular stroke	414	2.21	.70	5.77	3.89	3.37	2.12
**Pulmonary**							
Asthma	1876	2.55	.71	2.24	1.67	1.66	1.63
Chronic bronchitis	1460	2.50	.70	3.10	1.65	1.48	1.38
**Neurologic**							
Epilepsy	284	2.69	.74	1.70	1.10	1.03	1.12
Migraine	3280	2.68	.69	1.83	1.42	1.33	1.38
*Parkinson's disease*	*20*	*1*.*75*	.*44*	*8*.*59*	*15*.*66*	12.18	12.56
**Endocrine**							
Thyroid	788	2.47	.69	3.12	2.01	1.92	1.65
Diabetes	482	2.28	.67	4.68	3.07	3.10	2.30
**Renal**							
Kidney stone	1113	2.58	.69	2.35	1.52	1.59	1.16
**Gastrointestinal**							
Liver disease	393	2.49	.73	2.80	1.34	1.17	1.11
Ventricular ulcer	1026	2.42	.70	3.83	2.12	2.08	1.72
Duod. ulcer	953	2.48	.69	3.09	1.71	1.70	1.52
**Cancer**							
Cancer survivor	790	2.51	.72	2.80	1.69	1.61	1.19
**Dermatological**							
Psoriasis	1636	2.72	.70	1.58	1.25	1.21	1.22
Atopic eczema ^b^	2325	2.85	.70	1.08	.88	.84	.99
Hand eczema	3450	2.81	.69	1.26	1.06	1.06	1.09
**Miscellaneous**							
Pollen allergies ^c^	2851	2.88	.71	.97	.72	.71	.81
Osteoporosis	350	2.11	.57	9.29	4.70	4.63	2.56
*Arthritis*	*730*	*2*.*18*	.*60*	*3*.*05*	*2*.*17*	1.99	1.99
*Rheumatoid arthritis*	*162*	*1*.*99*	.*64*	*4*.*63*	*3*.*09*	2.94	2.99
Food allergies	1769	2.77	.71	1.41	1.14	1.13	1.29
Hypersensitivity	3184	2.71	.70	1.75	1.25	1.13	1.20
Fibromyalgia	1872	2.23	.62	8.19	6.78	6.07	5.93
Urinary incontinence	*409*	2.15	.64	*2*.*88*	*1*.*71*	1.41	1.40
Glaucoma	*141*	2.25	.66	*1*.*75*	.*82*	.99	.96
Cataract	*421*	2.27	.63	*1*.*77*	*1*.*19*	1.05	1.01

^a^ Independent samples Mann-Whitney U test show significant lower SRH levels for all comorbid conditions (p <.001) except for

^b^p =.086 and

^c^ p =.494.

^d^ Full contextual model (n = 17,475), Log likelihood = -14876.112, Nagelkerke R^2^ =.160, χ^2^ (32) = 399.05, P <.001, Contextual model over 70y (n = 1094) is marked by italics, Log likelihood = -909.06, Nagelkerke R^2^ =.180, χ^2^ (35) = 414.47, P <.001.

^e^ = reference category

#### Mental health

Tromsø 5 and 6 used the Hopkins Symptom Check List (HSCL-10), which is a self-reported symptom inventory comprising ten items that are representative of the symptom configurations commonly observed among outpatients [33]. Tromsø 4 used the mental health index (MHI) based on seven questions on different dimensions of mental distress. MHI was partly derived from HSCL and the General Health Questionnaire (GHQ). The scales were compared in the Cohort Norway panel (CONOR) that included both HSCL-10 and MHI. It was found that MHI was highly correlated to HSCL-10, with the conclusion that the Mental Health Index composed of the seven questions on mental distress is a valuable and valid tool in epidemiological research. For significant symptoms we use the recommended cut-off of 1.85 for HSCL-10 and 2.15 for MHI [34, 35].

### Statistical analysis

We used STATA v14 for the entire analysis. Steps 1–4 are based on the reference population, while Step 5 is based on the validation populations. Step 6 compares predicted and observed SRH in both populations.

#### Step 1: Describing the impact of individual comorbid diseases on SRH

We used SRH from Tromsø 4 as the outcome of interest when estimating the effect of the comorbid disease. We show count, mean, and standard deviation for all variables. We compared different SRH levels for gender, comorbid condition, age, and mental health symptoms, utilizing non-parametric statistics (Mann-Whitney and Kruskal-Wallis).

#### Step 2: Identifying relevant predictive factors and estimating their effect on SRH

We utilized ordinal logistic regression to assess the relationship of important potential predictive variables to SRH in the reference population. Such models can be used to estimate the odds of being at or above a given threshold across all cumulative splits, considering the effects of a set of explanatory variables [36]. Proportional odds models focus on cumulative probabilities rather than probabilities for discrete categories. The odds ratios are the odds of scoring at lower levels of SRH for those *with* the disease compared with those *without* the disease. An OR>1 is thus associated with a probable *negative* impact on SRH.

The models were built stepwise, starting with *the unadjusted models* that estimate the total effect of each comorbid disease, age, mental health, and gender. *The base model* controls each comorbid disease against all others. *The mental health model* controls for mental health. *The full contextual model* controls for all variables including age and gender.

#### Step 3: Establishing the comorbidity index

We created a scoring system based on the estimated ORs. We excluded comorbid disease with OR≤1.2. The assigned weight is the OR for the condition rounded off to the closest natural number. The health impact index score (HII) is the total of the weighted score for every condition the patient has. We calculated the HII for the reference and validation population and created the comorbidity groups “Not ill” (HII = 0), “Mildly ill” (HII = 1–2), “Moderately ill” (HII = 3–5) and “Seriously ill” (HII≥6).

#### Step 4: Calculating the predicted SRH in the reference population

We utilized ordinal regression to analyze how HII affects SRH when controlling for mental health symptoms, age, and gender. To predict SRH, we include all the factors identified in Step 2. The model, therefore, equals the full contextual model but replaces the specific medical conditions with HII. It was given by:

$$logit\;\left(Y\leq i\right)=\;(Z_{i}+\;\beta_{2}hii+\;\beta_{3}mhi+\;\beta_{4}age+\;\beta_{5}sex),\;\;i=1,2,3$$

We report the category thresholds (logit/Z_i_) and location (β_n_) values. We can now calculate each subject’s cumulative odds by filling in the variables and the coefficient into the model equation or we can use postestimation in STATA to calculate the category probability for the reference population. Cumulative odds are *EXP* (*Z*_*i*_ + *β*_2_*hii* + *β*_3_*mhi* + *β*_4_*age* + *β*_5_*sex*). The cumulative proportions are 1/(1+cumulative odds). Category probability is the difference between the cumulative proportions at each level against the level above. The estimated average for each subject is the total of the cumulative proportions [36].

#### Step 5: Calculating predicted SRH in the validation population

For the validation population, we need to calculate the category probability without using the observed SRH. We utilized the model from the reference population to predict category distribution and estimated SRH. We calculated the predicted category and SRH using the coefficients from step 4 and the variables from the FHI panel for each subject.

#### Step 6: Comparing the predicted and observed SRH in both populations

We checked the goodness of fit by comparing the observed SRH to the distribution of the predicted variables, with the statistical significance of the test summarized by a p-value utilizing Wilcoxon signed rank matched pairs [37]. We used Spearman’s correlation to compare the association of SRH to both HII and the Charlson Comorbidity Index (CCI). These are non-parametric statistics based on ranked data and are useful to minimize the effects of extreme scores [34]. Spearman’s correlation was performed for the whole sample and for a subsample including individuals with at least one identified comorbid disease (HII>0 for the HII and CCI>0 for the CCI). The analytical goal was to determine whether the association remained stable in those subjects that the indexes identified with a comorbid condition.

### Ethics

The Norwegian Data Protection Authority and the Regional Committees for Medical and Health Research Ethics North Norway approved the Tromsø Study.

## Results

### Determining impact and interaction of comorbid disease

Table 1 shows count, mean and standard deviation of SRH for each age group, gender, and comorbid disease. Although the effect of comorbid disease differs in severity, SRH levels were significantly lower in the presence of a comorbid disease (p <.001), except for pollen allergies (p = .494) and atopic eczema (p = .086). The youngest age group had the highest score, with a successive and significant decline in the SRH distribution with increasing age (p <.001). The presence of mental health symptoms had a significant negative effect on SRH with increasing effect depending on the severity of symptoms (p <.001).

The impact on SRH is reported as Odds Ratio (OR) and conditions such as angina (OR 8.9) and osteoporosis (OR 9.3) had the highest impact on SRH while pollen allergies (OR .97) and atopic eczema (OR 1.1) had low impact. Comparing the unadjusted OR with the base model showed that the OR was lower when controlling for each comorbid disease against all others. Diabetes had an OR of 4.7, but it is 3.1 when controlling for other diseases, meaning that comorbid disease explained part of its total effect. Glaucoma had an OR at 1.8 unadjusted and 0.8 adjusted. This means that even though we initially found that all comorbid disease was associated with a lower SRH, this association was not always explained by the condition itself but by other comorbid diseases.

For most comorbid conditions, the negative effect was weaker when controlling for mental health. This means that mental conditions explain part of the negative impact. Some conditions were not affected by mental conditions, e.g. asthma (OR 1.7) and diabetes (OR 3.0). We also see that significant symptoms have a large negative effect on SRH (OR 17.7), controlled for all comorbid conditions.

*The full contextual model* controls for all variables including age and gender. The main effects on SRH were comorbid conditions, mental health, and age. Here, angina, myocardial infarction, stroke, and osteoporosis had a much lower OR, mainly because age explains part of the effect. The effect of age implies that the persons react differently to illness with increasing age. Age had a negative impact on SRH with an OR 8.9 for subjects older than 80 years. Although men on average reported higher levels of SRH (2.9 vs. 2.8 for women), the contextual model indicated that males were associated with a 5% negative impact (OR 1.05) on SRH as compared to females in the fitted model. Significant mental health symptoms were associated with a substantial negative effect (OR 20.8).

### Establishing the comorbidity index

Table 2 shows the Health Impact Index with weights assigned to each comorbid condition based on the full contextual model. The HII is the total sum of the assigned weights for all the conditions the subject has. We observed two contrasts to indexes based on death as outcome: Diseases that were not classified as comorbid conditions according to Charlson et al. were significantly related to SRH [4, 5]. Diseases were also weighted differently, indicating that mortality does not explain comorbid effect on SRH.

**Table 2:** Weighted health impact of comorbid physical conditions.

Comorbid conditions	Assigned weight
Hypersensitivity	1
Psoriasis	1
Food allergies	1
Chronic bronchitis	1
Migraine	1
Urinary incontinence	1
Duod. ulcer	2
Asthma	2
Thyroid	2
Ventricular ulcer	2
*Arthritis*	2
Myocardial infarction	2
Cerebrovascular stroke	2
Diabetes	2
Osteoporosis	3
Angina	3
*Rheumatoid arthritis*	3
Fibromyalgia	6
*Parkinson's disease*	13

Assigned weights for each condition the participant has. The total equals the score. A patient with angina (3) and diabetes (2) will have a full contextual score of 3+2 = 5.

### Validating the comorbidity index

The health impact index was significantly related to how well people scored their SRH (R_S_ = -0.360, p <.001). We also found that SRH was significantly related to the Charlson Comorbidity Index (R_S_ = -0.250, p <.001). However, by excluding all individuals classified as “not ill”, the relation between SRH and HII was strengthened (R_S_ = -0.421, p <.001), while the relation between SRH and Charlson’s index was attenuated (R_S_ = -0.141, p <.001). It shows that CCI does not correctly assess SRH, especially concerning the presence of comorbid conditions.

**Fig 1:**
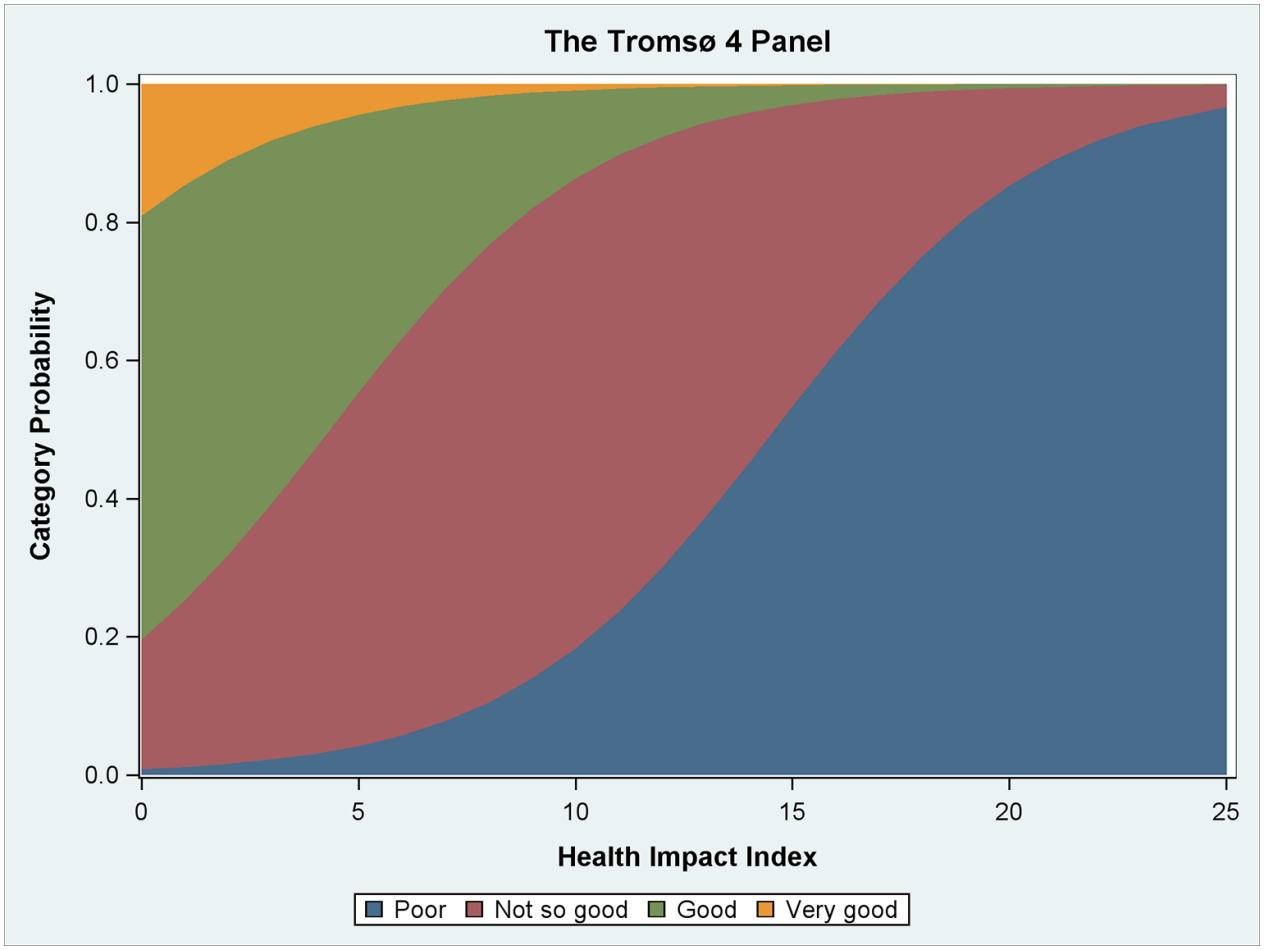
Category probability across HII score. The figure shows how increased comorbid strain was associated with a probability of scoring at lower levels on the SRH scale.

Table 3 shows the results from the ordinal regression model in Step 4, using HII as variable instead of the specific medical diagnosis. We see that HII had a negative effect on SRH in the fitted model. Fig 1 is based on the ordinal regression model and visualizes how increasing HII implied reduced SRH levels. We also observe that mental health and age are significant factors. We find no significant gender differences.

**Table 3:** The results from the ordinal regression model showing the impact of comorbid disease on SRH controlled for mental health symptoms, gender, and age.

	Full contextual model with HII					
	Coef.	Std. Err.	z	P>z	[95% Conf. Interval]	
**Health Impact Index**	-0.249	0.006	-42.030	<.001	-0.260	-0.237
**Mental Health Index**	-1.915	0.036	-53.550	<.001	-1.985	-1.845
**Age**	-0.048	0.001	-48.270	<.001	-0.049	-0.046
**Gender**	-0.036	0.027	-1.340	0.180	-0.088	0.017
**/threshold1**	-10.566	0.110			-10.781	-10.351
**/threshold2**	-6.682	0.083			-6.844	-6.520
**/threshold3**	-3.330	0.073			-3.473	-3.188

Log likelihood = -22364.373, LR chi2(4) = 9817.75, p <.0001

**Fig 2:**
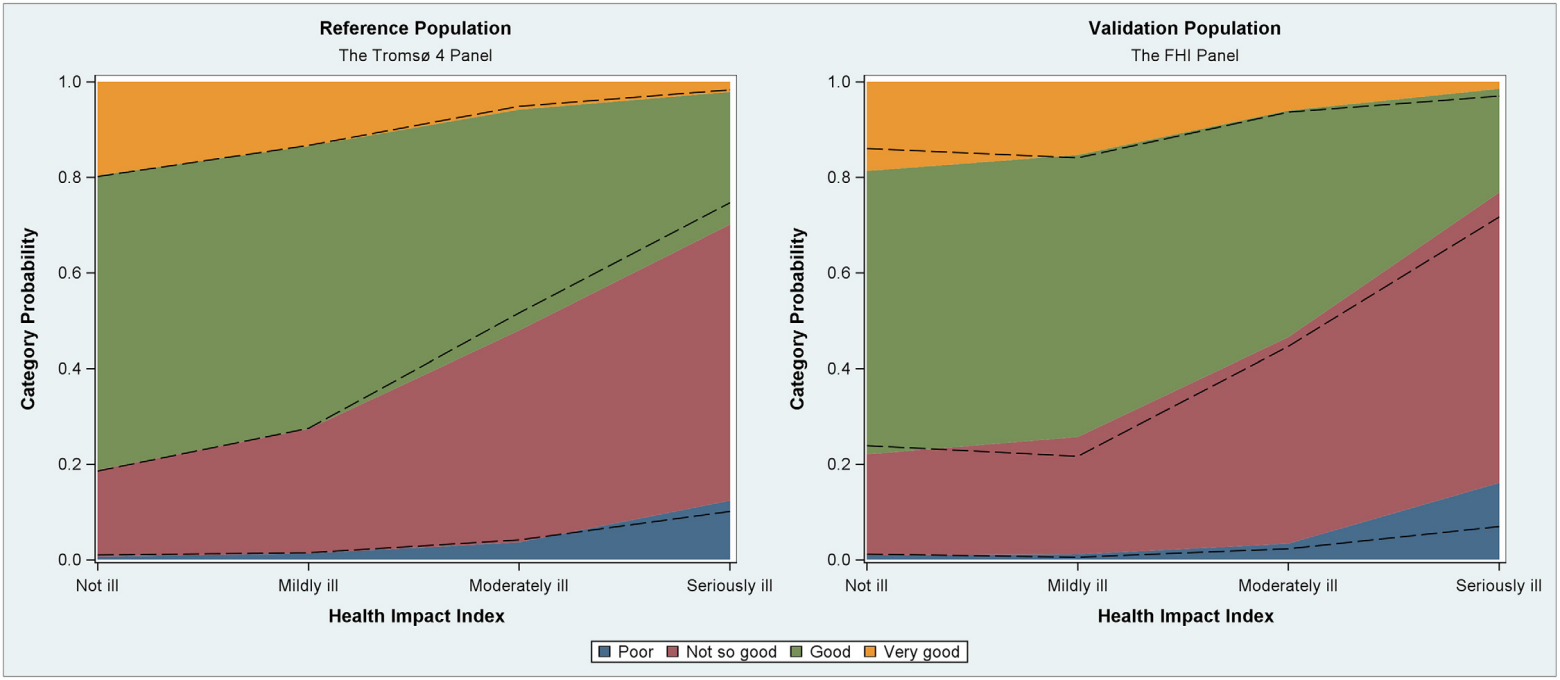
Predicted category distribution based on the ordinal regression model in the reference populations versus the health impact score. Dashed lines represent the observed category distribution. Visual inspection shows that the observed category distributions are similar to those predicted.

**Fig 3:**
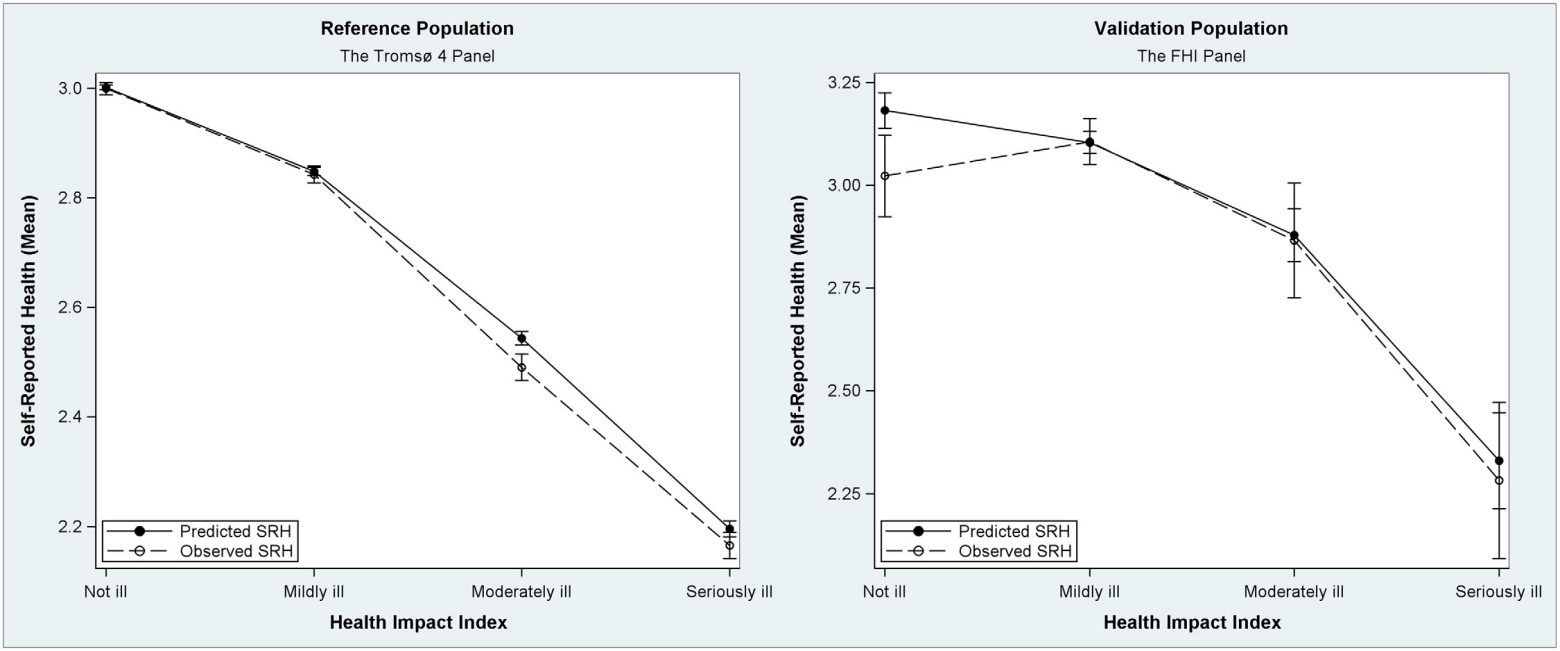
Comparison of observed and predicted category distribution. Dashed lines represent the observed SRH. The figure shows how SRH decreases with increasing comorbidity, and the difference between predicted and observed SRH.

Table 4 shows the comparison of the estimated and reported SRH from Steps 4–6 of the analysis. Figs 2 and 3 visualize the comparison of observed Self-Rated Health and predicted Self-Rated Health. We found no significant difference at a significance level of .05 for either the FHI panel (z = 1.007, p = 0.314) or the Tromsø 4 panel (z = 1.254, p = 0.210). This means that the model predictions are not significantly different from the observations.

**Table 4:** Comparison of observed and predicted Self-Rated Health.

	Tromsø 4 panel ^a^			FHI panel ^b^		
	Observed SRH (Mean)	Predicted SRH (Mean)	Count	Observed SRH (Mean)	Predicted SRH (Mean)	Count
**Not ill**	3.00	3.00	12.982	3.02	3.18	176
**Mildly ill**	2.84	2.85	6.881	3.11	3.10	482
**Moderately ill**	2.49	2.54	2.824	2.87	2.88	97
**Seriously ill**	2.17	2.20	2.552	2.28	2.33	39

^a^. Wilcoxon signed rank matched pairs: z = 1.254, P = 0.2100

^b^. Wilcoxon signed rank matched pairs: z = 1.007, P = 0.3137

## Discussion

We found that disease had a varied but consistent negative impact on SRH. The regression models show how part of this effect was explained by comorbidity as well as age, gender and mental health symptoms. Comorbidity, increasing age and presence of mental health symptoms were associated with the probability of lower SRH. Females scored lower on SRH than men. Based on these findings, we constructed an index (HII) that estimates the comorbid strain on SRH, controlling for gender, age, mental health symptoms and other diseases. When applying the index to both the reference and validation population, our estimation and the observed SRH showed no significant differences.

Our findings concur with other studies that focus on the association between SRH and bio-medical variables. Specific medical conditions represent one crucial biological pathway that affects SRH; however, even when controlling for comorbid disease, SRH remains an independent predictor of mortality [14]. A second biological pathway that has been debated is the associations between SRH and more objective health measures and known health risk factors (e.g. resting heart rate, blood pressure, cholesterol, BMI, and endocrine measures). Although the effect of SRH attenuates when such variables are controlled, SRH remains an independent variable for future health outcomes [15, 16, 20, 38–40]. The literature on self-reported health suggests that self-ratings of health are produced in a cognitive process that is inherently subjective and contextual but that the basis of self-rated health lies in the biological and physiological state of the individual organism, which might explain why it predicts mortality and other health outcomes [14].

We know from our literature review that the association between SRH and future health outcomes becomes stronger when combined with other health measures such as specific medical conditions, biological markers as well as mental health and social context [12, 14–16]. The contribution of this study is to provide a method to include specific medical conditions with a variable that is related to comorbid strain on self-reported health.

Current methods, e.g. CCI, use mortality as a measure of general health status. The alternative approach, reported in this article, is to classify patients with comorbid disease according to the impact on SRH. Both methods are concerned with health loss due to comorbid disease, but the health impact index (HII) estimates the comorbid effect on SRH as the outcome of interest instead of death. HII has thus the potential to measure health along a broad spectrum of health-related variables. This implies not only that other diseases come into focus but also that the severity is weighted differently from mortality-based methods.

We found that CCI was also associated with SRH; however, the association was attenuated when focusing on comorbid disease. The reason CCI predicts SRH is that it identifies some conditions that have a negative impact on SRH. There will consequently be a difference between those with CCI = 0 (n = 19,586) and all those without (n = 5,653). CCI was designed to control for comorbid conditions when survival/mortality is the outcome of interest. Consequently, it cannot be expected to address the problem of comorbid disease as a confounder with other outcomes.

The methodological approach represented by HII is easy to use and to assess presence and severity of comorbid disease. For longitudinal designs, patients could enter with comorbid conditions or the conditions may arise during the time of follow-up. Our study uses HII as a continuous variable to predict SRH. For most clinical studies, it should be possible to stratify the population into 2–5 groups based on HII. In a general population, participants classified as ‘not ill’ (HII = 0) could serve as the reference group. In follow-up studies, it is an alternative to stratify by change/no change in HII.

One advantage of an SRH-based index is that it allows comparison of conditions that do not necessarily translate into mortality. It is, therefore, possible to pursue health impact on social and psychological variables. We found significant associations between SRH and mental health. HSCL-10 is particularly sensitive to anxiety and depression [33, 34]. It should be possible to construct a similar comorbidity index for mental health conditions.

Comorbidity indexes have previously been applied by health planners and policy makers to estimate risk and future health costs [6, 8, 9]. SRH is a well-established, simple, patient-centered tool for the assessment of illness in the context of multiple chronic disease diagnoses. It has also been suggested that SRH should guide clinicians when prioritizing patients, based on its ability to predict future health outcomes and use of health services [1]. However, since SRH is self-reported, it might become biased if patients know their responses will be connected to individual rights and access to health services. The use of HII could assist clinicians and health policy makers to prioritize at both the group and individual level, e.g. those registering with three or more diseases and/or with poorer self-rated health may warrant further assessment and intervention to improve their physical and subjective health [1]. To our knowledge, it is not a systematic practice to assess comorbid health strain. The next step would be to show that it has clinical significance in a follow-up study.

A balanced health assessment would lead to emphasis on of the phenomenon of comorbidity in clinical care, epidemiology, and health services [2], because it allows for comparison of different phenomena that do not necessarily translate into mortality, but still translate into health [12, 14].

### Limitations

Although this study, to our knowledge, is the first to validate a comorbidity index based on SRH, it could be further developed. The Tromsø Study contains a wide variety of diagnoses, but lacks information on degree of severity. It is particularly relevant to cancer diagnoses. The low score of the cancer survivors (2.8) is interesting; despite being the “emperor of all maladies” [41] it has a rather small impact on SRH. One explanation is that the condition includes all cancer diagnoses, including fully recovered/survivors. In contrast, fibromyalgia, osteoporosis, and angina are not conditions associated with high mortality, and yet they influence the SRH to a greater degree, showing that mortality risk and SRH are separate dimensions of health. In a preliminary analysis of those who died within two years and 20 years of the study, the SRH was 2.2 and 2.4, respectively. It is comparable to the myocardial and vascular disease group; however, surviving (>20 years) with the diagnosis still lowers the SRH (2.7). This indicates that being diagnosed with cancer in itself could belong in the index but we need data that differentiates the severity of the diagnosis.

Parkinson’s disease is a limiting case. It is the strongest finding with an OR of 12.6. Even though it is based on just 20 cases, it is highly significant (p <.001) but with a wide confidence interval (3.0–53.0). Steps 3–6 were run with and without Parkinson. It did not affect any of our estimates. Based on the confidence interval, it should at least acquire a weight of 3. Accordingly, we have kept it in the index but more data would be preferable.

All ordinal models were tested for proportional odds (PO) assumption with a 1000 random sample and with separate binary, ordinal regression models for each cutoff point. PO implies that the OR is the same across the categories. PO is a conservative criterion and sensitive to a large number of variables, many participants, and interactions between variables [36] and yet the assumption was fulfilled for all except for myocardial infarction and angina. Partial proportional odds models were run for these conditions. The OR at the middle threshold represents the median value for both.

Power calculations for the validation population show that with a power of 80% at an alpha level of .05, the test is sensitive to a delta of .10 [42]. The reported mean difference was .025 that is within this limit with a good margin.

## Conclusion

Comorbid strain on health can be measured by using either survival or self-reported health as outcome of interest. CCI estimates comorbid mortality while HII estimates the comorbid impact on SRH. HII outperforms CCI in predicting SRH, especially in the group with a comorbid disease. We conclude that the health impact index should be used when self-reported health is not available, and well-being or quality of survival/life is the outcome of interest. The result of utilizing SRH as the basis for the impact of comorbidity on health could be population health research that does not focus solely on health as absence of disease but also on the well-being of living with infirmity and disease.
